# The potential impact of clindamycin on neurosurgery patients: a randomized controlled trial

**DOI:** 10.1007/s10787-025-01810-1

**Published:** 2025-06-23

**Authors:** Lobna w. Alam El-Din, Noha A. El-Bassiouny, Wael M. Khedr, Rehab H. Werida

**Affiliations:** 1 Clinical Pharmacy and Pharmacy Practice Department, Alexandria University Main Teaching Hospital, Alexandria, Egypt; 2 Clinical Pharmacy & Pharmacy Practice Department, Faculty of Pharmacy, Damanhour University, Damanhour, El-Behirah Egypt; 3 Neurosurgery Department, Faculty of Medicine, Alexandria University, Alexandria, Egypt

**Keywords:** Ceftriaxone, Clindamycin, Neuroprotection, Neurosurgery, Complications, Neurological disorders, Neuron apoptosis, Central nervous system infections, Neuron-specific enolase (NSE), Neurotensin (NT), Glasgow coma scale (GCS), SOFA score

## Abstract

**Purpose:**

The study investigates whether adding clindamycin to neurosurgery patients’ as a postsurgical management regimen improves recovery, provides neuroprotection, and prevents neurological complications. Neuron-specific enolase (NSE) and neurotensin (NT) were measured as biomarkers of inflammation, brain damage, and neuronal apoptosis.

**Methods:**

Patients were randomly assigned into two groups (n = 22 each) to receive the standard management plus either ceftriaxone (2 g / 12 h) or plus ceftriaxone and clindamycin (900 mg/8 h) as a combination therapy for seven days.

**Results:**

NSE serum levels in the clindamycin and control group on day 3 were (10.01 ± 1.64) versus (23.77 ± 11.75), respectively (*p* = 0.0001). NT serum levels in the clindamycin and control groups on day 3 were (4.5 ± 2.8) versus (8.29 ± 7.97), respectively (*p* = 0.0418). Glasgow Coma Scale (GCS) on day 3 was (14.32 ± 1.13) versus (14.23 ± 1.31) in the clindamycin and the control groups, respectively, (*p* = 0.724). SOFA score assessed on day 3 (5 (22.7%)) and (1 (4.5%)) had grade 1, (15 (68.25)) and (14 (63.35)) had grade 2, (1 (4.5%)) and (5 (22.7%)) had grade 3, (0 (0.0%)) and (1 (4.5%)) had grade 4, and (1 (4.5%)) and (1 (4.5%)) had grade 5 in the clindamycin and control groups, respectively.

**Conclusion:**

Adjunctive use of clindamycin might be a novel option that reduces secondary neurological injury/damage after neurosurgeries. Further and more extensive clinical trials are warranted to confirm the findings.

## Introduction

Neurosurgical procedures are critical interventions to manage and correct disorders of the central nervous system (CNS), including conditions affecting the brain, spinal cord, and peripheral nerves. Globally, neurological disorders and injuries affect approximately 22.6 million per year, with 13.8 million requiring surgical interventions (Dewan et al. 2018). The primary complications after neurosurgery are neuronal apoptosis and CNS infections, which may cause long-term neurological sequelae in addition to significant morbidity and mortality. They are mainly a consequence of the direct toxicity of bacterial hemolysins on neurons and the host’s systemic inflammatory response, which may lead to leukocyte extravasation into the subarachnoid space. The surgeries may cause acute brain injury that triggers a series of biochemical events at the cellular level called the ischemic cascade, leading to a loss of integrity and function of the neurons, consequently decreasing the chance of functional recovery (Waraich and Ajayan 2024). Furthermore, brain edema, vasculitis, or stimulation of resident microglia within the CNS by bacterial compounds may occur (Moujalled et al. 2021; Ethiraj et al. 2014).

Sustained postsurgical inflammation, caused by traumatic incision, infection, or autoimmune response, triggers intrinsic and extrinsic apoptotic pathways in neurons, resulting in neuronal damage. Activated microglia and astrocytes release proinflammatory cytokines (IL-1β, TNF-α, IL-6), reactive oxygen species (ROS), and nitric oxide (NO), all of which can disrupt neuronal function, damage DNA, and impair mitochondrial activity (Nau and Eiffert 2002; Wright and Collier 1976; El-Ansary et al. 2013).

In animal models, rifampicin, clindamycin, and daptomycin were found to reduce inflammation, mortality, neuronal injury, and neurological long-term sequelae. Compared to β-lactam antibiotics, these antibiotics better prevent microglia activation and protect the nervous tissue (Tan et al. 2015). In particular, rifampin has been demonstrated to be neuroprotective in animal models of both permanent and transient focal cerebral ischemia. However, debate remains as to whether rifampin activates glucocorticoids and whether its beneficial effects result from inhibiting the release of proinflammatory/toxic bacterial products or direct immunosuppression (Esposito et al. 2004). In contrast, clindamycin’s neuroprotective properties have been confirmed in preclinical studies despite its lack of immunosuppressive action.

Clindamycin, an antibiotic that is a chemical derivative of lincomycin, acts against anaerobic Gram-negative and aerobic Gram-positive bacteria. Its primary mechanism of action involves the binding of clindamycin to the 50S ribosomal subunit that inhibits bacterial protein synthesis (Picardi et al. 1975). Although the penetration of clindamycin is considered poor, preclinical studies proved that cerebrospinal fluid levels averaged 20.5% of paired serum concentrations and were higher than the concentrations needed to inhibit most Gram-positive bacteria (Patel et al. 2020).

Furthermore, previous studies revealed that combining ceftriaxone with clindamycin improves survival by suppressing infection, inflammatory response, oxidative stress, and neutrophil infiltration (Iacob et al. 2007). A previous study performed on mice using a protein synthesis inhibitor antibiotic, which belongs to the same mechanism of action and antibiotic class of clindamycin, demonstrated a decreased mortality and neuronal injury in pneumococcal meningitis compared with the β-lactam antibiotic ceftriaxone (Nau et al. 1999; Bӧttcher et al., 2000). Adding clindamycin to β-lactam therapy was associated with reduced mortality (15% vs. 39%) in another retrospective study performed on 84 adult patients with severe invasive group A streptococcal (GAS) infection (Carapetis et al. 2014; Barker 2007).

Neuron-specific enolase (NSE) is a glycolytic enzyme predominantly located in neurons and neuroectodermal cells, serving as a marker of neuronal damage. NSE is a promising marker of brain damage and recovery after neurosurgery. Its increased concentration can be measured in the cerebrospinal fluid (CSF) and peripheral blood after neuronal damage, which provides a reliable laboratory indicator of the degree of brain cell damage and may allow for early outcome prediction (Lima et al. 2004; Lee et al. 2021; Haque et al. 2018).

Clindamycin reduces proinflammatory cytokines such as TNF-ά and IL1-β, which may lead to a reduction in neuronal inflammation, potentially resulting in decreased neural stress or damage, thereby lowering NSE levels. Anti-inflammatory cytokines, such as IL-10, are influenced by clindamycin, promoting its neural protection. This protective effect could contribute to standardizing NSE levels (Kishi et al. 1999; Rodrigues et al. 2023, 2020,b).

Neurotensin (NT) is a 13-amino-acid peptide acting as a neurotransmitter and hormone. NT acts as a proinflammatory cytokine, found both in and outside the nervous system, serving as a marker of inflammation. It is an inflammatory associated rather than an inflammatory modulator, as it can affect immune response and cytokine release. NT has no specific normal range, since it is a relatively new marker. According to some studies, NT in neurodegenerative disease and trauma might be elevated due to a proinflammatory state or neurological damage. Neurosurgery patients with elevated NT levels are suspected due to response to brain injury or inflammation, blood-brain barrier disruption, and systemic stress response (Castagliuolo et al. 1999; Huidobro-Toro and Yoshimura 1983).

NT was initially characterized as inducing inflammatory symptoms, including mast cell degranulation, vasodilatation, improved vascular permeability, phagocytosis of neutrophils, and enhanced directional migration (Goldman, Bar-Shavit, and Rameo, 1983; Dicou et al. 2004). Moreover, NT stimulates the production of interleukin-1 (IL-1) by activating alveolar macrophages (Lemaire 1988). Furthermore, an increase in NT is an indicator of glutamate increase, resulting in neurotoxicity and secondary neural damage (Ferraro et al. 2011; Boia et al. 2020).

This study aims to compare the effects of clindamycin on the neuroprotection and avoidance of neuronal complications caused after neurosurgery with the outcomes of standard therapy consisting of the β-lactam antibiotic ceftriaxone as a prophylactic postoperative antibiotic protocol. NSE and NT were used as primary outcomes to detect the prognosis and neural damage in the patients, whereas GCS scores and SOFA scores were used as secondary outcomes.

To date, clindamycin has not been clinically tested for neuroprotection, and no established evidence supports its use in this context. Its proposed neuroprotective role remains hypothetical and requires future investigation.

Therefore, this study would represent a novel therapeutic angle for repurposing an established antibiotic for CNS protection.

## Patients and methods

### Study design

This prospective, single-center, randomized, controlled, double-blinded study was conducted on neurosurgery patients at Alexandria University Main Hospital, Alexandria, Egypt. Institutional ethical approval of the study protocol was obtained (approval number: 123PP63). The study protocol was registered before patient enrollment at clinicaltrials.gov (NCT06068673). The study was performed according to the Declaration of Helsinki. Written informed consent was obtained from the patients or their legally authorized representatives before enrollment. Patients were monitored for five days, with a mortality follow-up over 21 days. Randomization was carried out using block randomization, ensuring equal numbers of participants in each block.

### Patients and intervention

Inclusion criteria**:**

Adult patients above 18 years who have undergone neurosurgery.

Exclusion criteria:

1. Allergy to studied medication.
2. Females with a positive pregnancy test.
3. Known congestive heart failure or ischemic heart disease.
4. Any injury that disturbs the examination (high cervical cord injury or locked-in syndrome could be a source of bias).
5. Renal failure with GFR lower than 60 mL/min.
6. Patients with unknown identity.

### Patient assessment and follow-up

All patients’ demographics, underlying diseases, medical history, surgical intervention, vital signs, and GCS values were assessed at baseline. Independent investigators evaluated the GCS scores blinded to treatment. Both outcome assessors and patients were blinded to the treatment administered.

### Study outcome

The study’s primary outcome was a difference between the two groups in mean NSE and NT serum levels at the peak on day 3. The study groups’ secondary endpoints are SOFA and GCS scores over the treatment period and mortality rate at 21 days.

### Biochemical analysis

Venous blood samples were collected from all patients to determine NSE levels after 24 h of the surgery, and NSE and NT after 72 h after randomization. According to previous studies, the peak level of markers occurs 72 h post-insult (Schoerkhuber et al., 1999; Motoyoshi et al. 2024). The serum was separated by centrifugation (3000 rpm for 10 min), isolated, and immediately frozen at -80°C. NSE level was assessed using enzyme-linked immunosorbent assay using a human NSE ELISA kit (Wuxi Donglin Sci & Tech Development Co., Ltd, Catalog No. DL-NSE-Hu), in addition to the NT level measured by enzyme-linked immunosorbent assay using a human NT ELISA kit (Wuxi Donglin Sci & Tech Development Co., Ltd, Catalog No. DL-NT-Hu) according to the manufacturer’s instructions.

### Statistical analysis

The sample size was calculated using G*Power software version 3.1.0 (Institut für Experimentelle Psychologie, Heinrich-Heine-Universität, Düsseldorf, Germany). A total sample size of 44 patients was estimated to have a power of 96% for detecting a medium-to-large effect size of 0.5 in the primary outcome measure. Statistical Package for Social Sciences (SPSS, RStudio) version 2.3.2. was used to analyze the data. The data were presented as numbers and percentages for the qualitative data, as mean, and standard deviations for the quantitative data with parametric distribution, and as median with interquartile range (IQR) for the quantitative data with the nonparametric distribution. Shapiro’s test was employed to test the normality of quantitative data. The chi-square test was utilized to compare two groups with qualitative data, and the Fisher exact test was used instead of the chi-square test when the expected count in any cell was less than 5. In contrast, an independent *t*-test was used to compare the two groups, using quantitative data and a parametric distribution. In contrast, the Mann–Whitney test was employed to compare the two groups using quantitative data and nonparametric distribution. A paired *t*-test was also utilized to compare two matched groups (before and after) using quantitative data with a parametric distribution. Conversely, a Wilcoxon rank test was used to compare two matched groups using quantitative data with a nonparametric distribution. The association between two quantitative datasets and parametric distributions was made using the Pearson correlation test. Spearman’s correlation coefficients were utilized to assess the significant relationship between two quantitative nonparameters in the same group. All p-values were two-sided, and a value of less than 0.05 was considered significant.

## Results

**Fig. 1:**
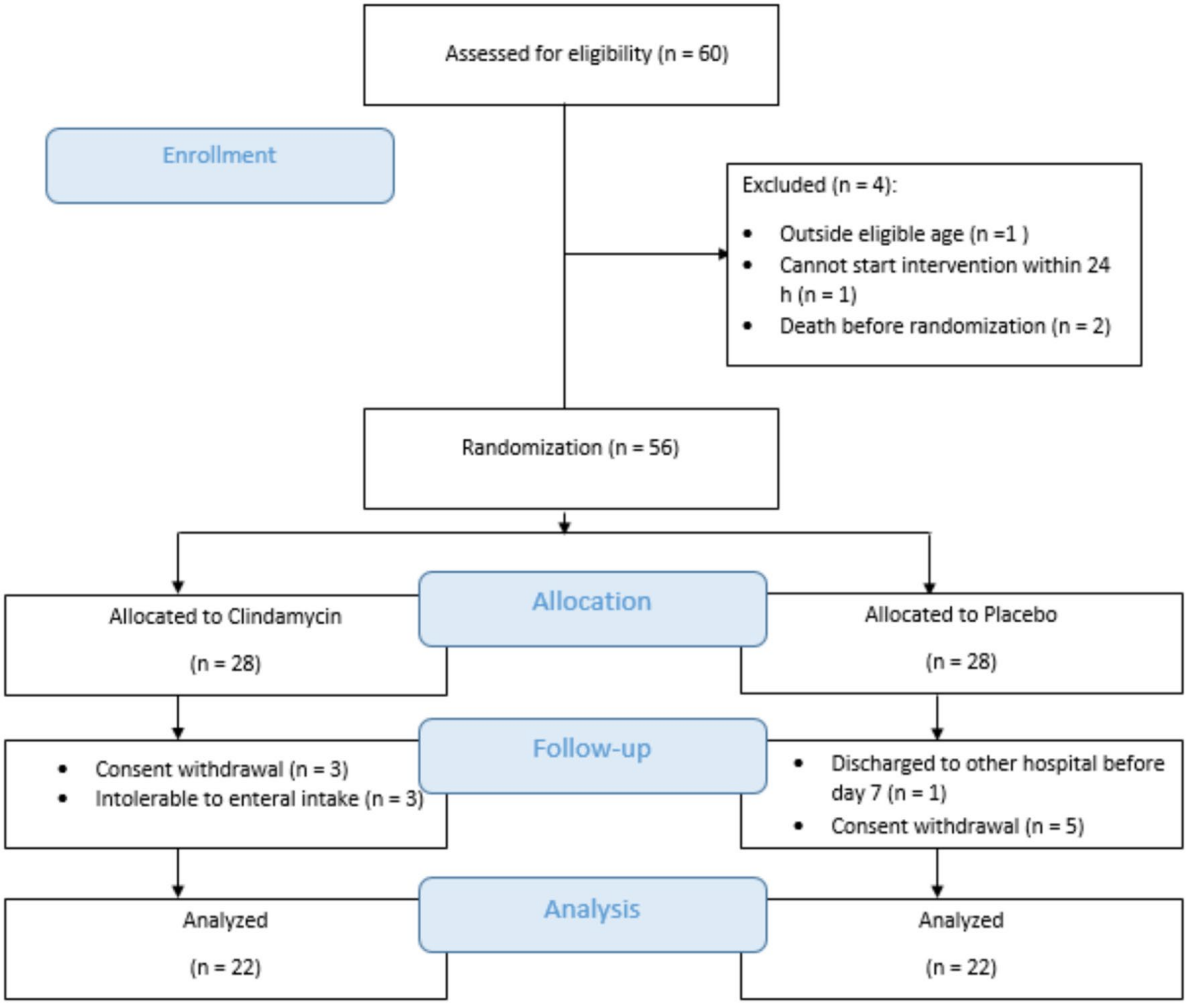
CONSORT flow diagram showing patient enrollment and follow-up throughout the study

The study was conducted between March 2023 and November 2023, involving 60 patients undergoing neurosurgery who were screened for eligibility. A total of 56 patients fulfilled the inclusion criteria and were randomly allocated to one of the study groups. Figure 1 demonstrates that 44 patients completed the study and were included in the final analysis. The baseline characteristics and physiological variables were balanced across the two groups. The type of surgery and severity of randomization were also similar between the two arms of the study (Table 1).

**Table 1:** Baseline characteristics of all enrolled patients

	Control group (n = 22)	Clindamycin group (n = 22)	p-value
**Sex**			
Male	11 (50.0%)	9 (40.9%)	0.762^†^
Female	11 (50.0%)	13 (59.1%)	
Age (years)	47.0 ± 14.7	41.7 ± 10.9	0.169^*^
BMI (kg/m2)	29.36 ± 9.58	30.24 ± 7.8	0.744^*^
**Comorbid diseases**			
**HTN**	9 (40.9%)	9 (40.9%)	< 0.01^†^
**HTN medication**			
ACEI	2	4	
ARB	0	1	
BB	4	6	
Diuretic	2	0	
**DM**	3 (13.6%)	7 (31.8%)	< 0.01^†^
**DM medications**			
Oral	1	6	
Insulin	0	1	
**Type of surgery**			
ETV	1 (4.5%)	1 (4.5%)	0.982^†^
Glioma	2 (9.1%)	2 (9.1%)	
Meningioma	9 (40.9%)	9 (40.9%)	
Posterior fossa	2 (9.1%)	2 (9.1%)	
Sellar lesion	3 (13.6%)	3 (13.6%)	
Temporal lesion	2 (9.1%)	2 (9.1%)	
TGN compression	0 (0.0%)	1 (4.5%)	
VPS	3 (13.6%)	1 (4.5%)	
VPS/hematoma	0 (0.0%)	1 (4.5%)	
**Vital signs**			
T (°C)	37.16 ± 0.35	37.06 ± 0.11	0.286^*^
HR (beats/min)	98.9 ± 5.43	98.14 ± 5.44	0.67^*^
RR (breath/min)	19.27 ± 1.86	19.5 ± 1.34	0.67^*^
SBP	125.80 ± 22.25	130.20 ± 15.71	0.233^*^
DBP	82.44 ± 11.64	82.80 ± 8.43	0.976^*^

Data are reported as mean ± SD, number, or as number (percentages) as appropriate

*p*-Values were obtained by ^†^ chi-square test or Fisher’s exact test as appropriate.

^*^ Mann–Whitney *U* test with significance set at *p* < 0.05.

BMI: Body Mass Index; HTN: Hypertension; ACEI: Angiotensin-Converting Enzyme Inhibitor; ARB: Angiotensin II Receptor Blocker; BB: Beta Blocker; DM: Diabetes Miletus; ETV: Endoscopic Third Ventriculostomy; TGN: Trigeminal Neuralgia; VPS: Ventriculoperitoneal Shunt; T: Temperature; HR: Heart Rate; RR: Respiratory Rate; SBP: Systolic Blood Pressure; DBP: Diastolic Blood Pressure

In each study group, NSE serum levels decreased over time compared to the baseline p = 0.3534) in the control group vs. (p = 0.0001) in the clindamycin group, as shown in Table 2.

**Table 2:** Patient outcomes during study days in the clindamycin and control groups

Parameter		Control group (*n* = 22)		*p*^*a*^	Clindamycin group (*n* = 22)		*p*^*a*^	*p*^*b*^
		Mean ± SD	Median (IQR)		Mean ± SD	Median (IQR)		
**NSE (ng/mL)**	24 h (baseline)	20.63 ± 10.44	15.85 (11.02)	0.3534^‡^	24.32 ± 12.11	17.15 (16.4)	0.0001^‡^	0.2850^*^
	72 h (peak)	23.77 ± 11.75	16.1 (19.05)		10.01 ± 1.64	10.16 (2.4)		0.0001^*^
**NT (pg/mL)**	72 h (peak)	8.29 ± 7.97	5.42 (7.14)		4.5 ± 2.8	4.66 (4.46)		0.0418^*^
**WBC (10^3/µL)**	72 h	18.77 ± 5.05	19 (4.7)		14.58 ± 5.49	13 (5.63)		0.0036
**SOFA**	Grade 1	5 (22.7%)			1 (4.5%)			0.105^†^
	Grade 2	15 (68.2%)			14 (63.3%)			
	Grade 3	1 (4.5%)			5 (22.7%)			
	Grade 4	0 (0.0%)			1 (4.5%)			
	Grade 5	1 (4.5%)			1 (4.5%)			
**GCS**	24 h	12.04 ± 1.001			12.5 ± 1.57			0.803^*^
	72 h	14.23 ± 1.31			14.32 ± 1.13			0.724^*^
	Day 7	14.45 ± 0.91			14.82 ± 0.39			0.0881^*^
**Hospital length of stay (days)**		7.77 ± 2.18			9.73 ± 2.69			0.0112^*^
**Death,** ***n*** **(%)**		1 (4.5 %)			0 (0.0 %)			1.0^†^

Data are reported as mean ± SD, Median (IQR) or as number (percentages) as appropriate

† chi-square test or Fisher’s exact test. * Mann–Whitney U test for comparisons between groups. ‡ T-test for Comparisons of groups over time

24 h: 24 hours after surgery; 72 h: 72 hours after surgery; NSE: Neuron Specific Enolase; NT: Neurotensin; WBC: White Blood Count; GCS: Glasgow Coma Score; SOFA score: Sequential Organ Failure Assessment; IQR: Interquartile range; SD: Standard deviation. ***p*a** within group. ***P*b** between both groups

**Fig. 2:**
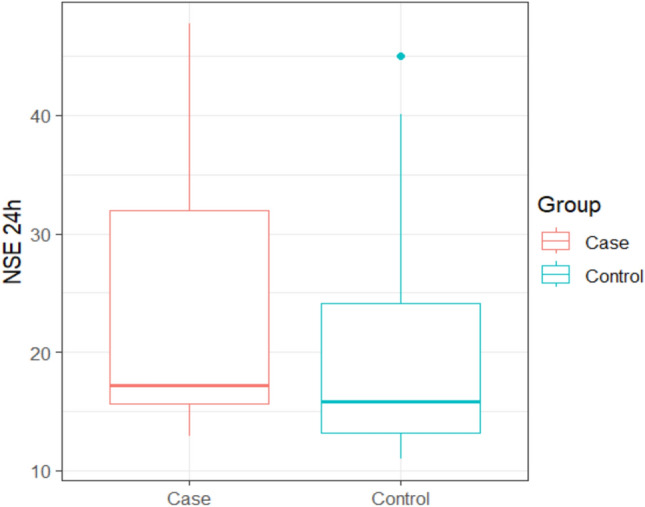
Box plot of NSE at 24 h between the two studied groups

Table 2 shows that neuron-specific enolase (NSE) at 24 h ranged between (12.9 – 47.7) with mean ± SD (24.32 ± 12.11) in the clindamycin group, while it ranged between (11 - 45) with mean ± SD of (20.63 ± 10.44) in the control group with no statistically significant difference between the two studied groups at (*p* =0.285), as seen in Fig. 2.

**Fig. 3:**
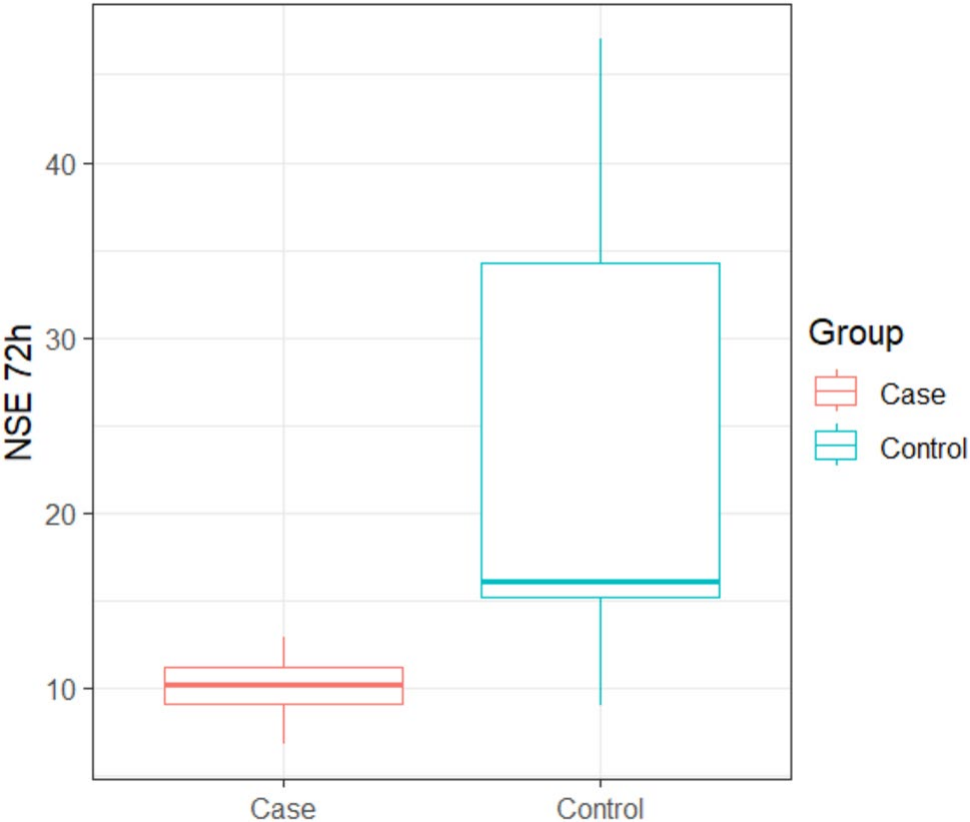
Box plot of NSE at 72 h between the two studied groups

**Fig. 4:**
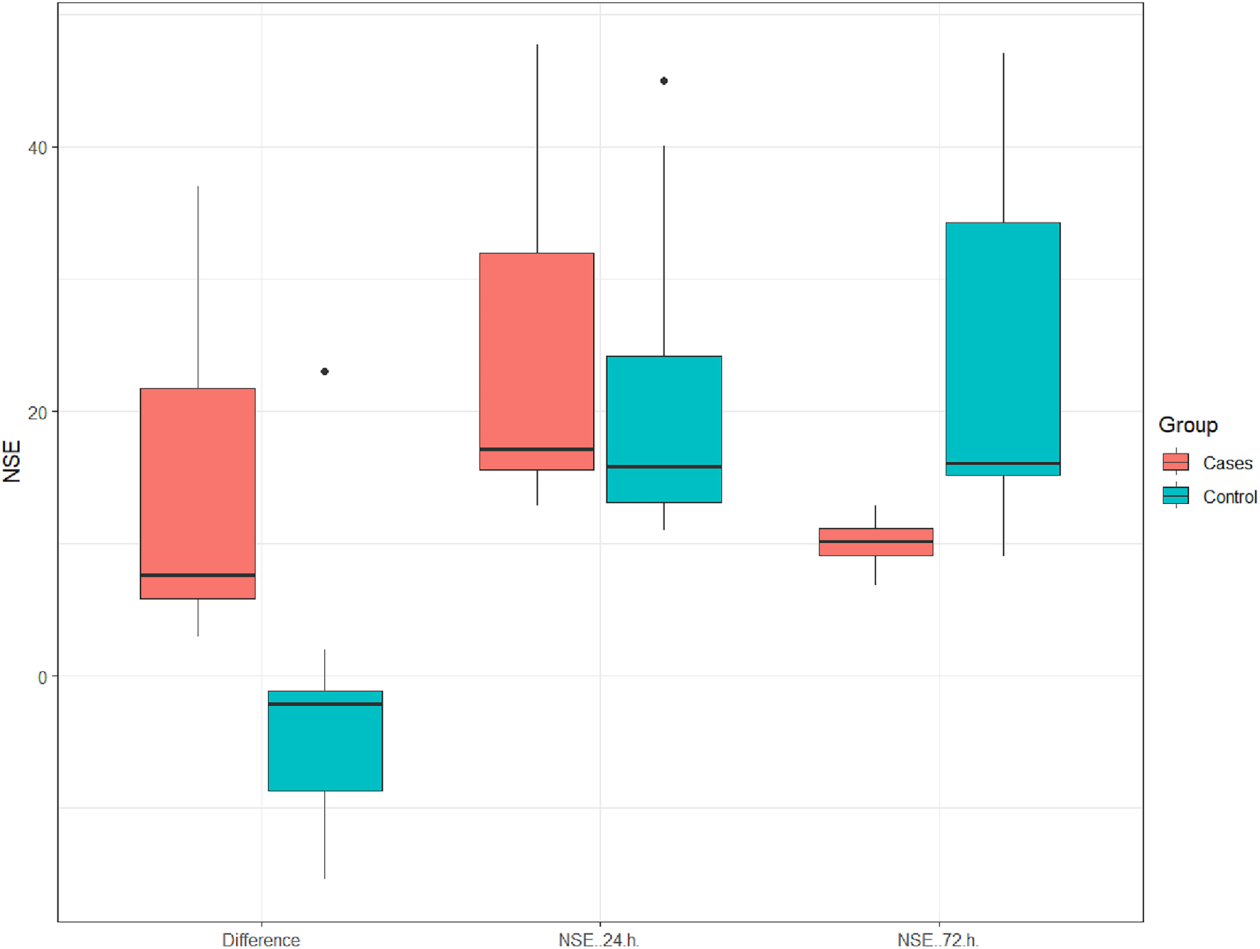
Cluster box plot for NSE (24 h, 72 h) between the two studied groups

NSE at 72 h ranged between (6.8 – 12.87) with mean ± SD of (10.01 ± 1.64) in the clindamycin group, while it ranged between (9 – 47) with mean ± SD of (23.77 ± 11.75) in the control group with a statistically significant difference between the two studied groups (p =0.0001), as shown in Table 2 and Fig. 3. In addition to, a comparison between NSE value at 24 h, 72 h, and their differences in the two groups, as shown in Fig. 4.

**Fig. 5:**
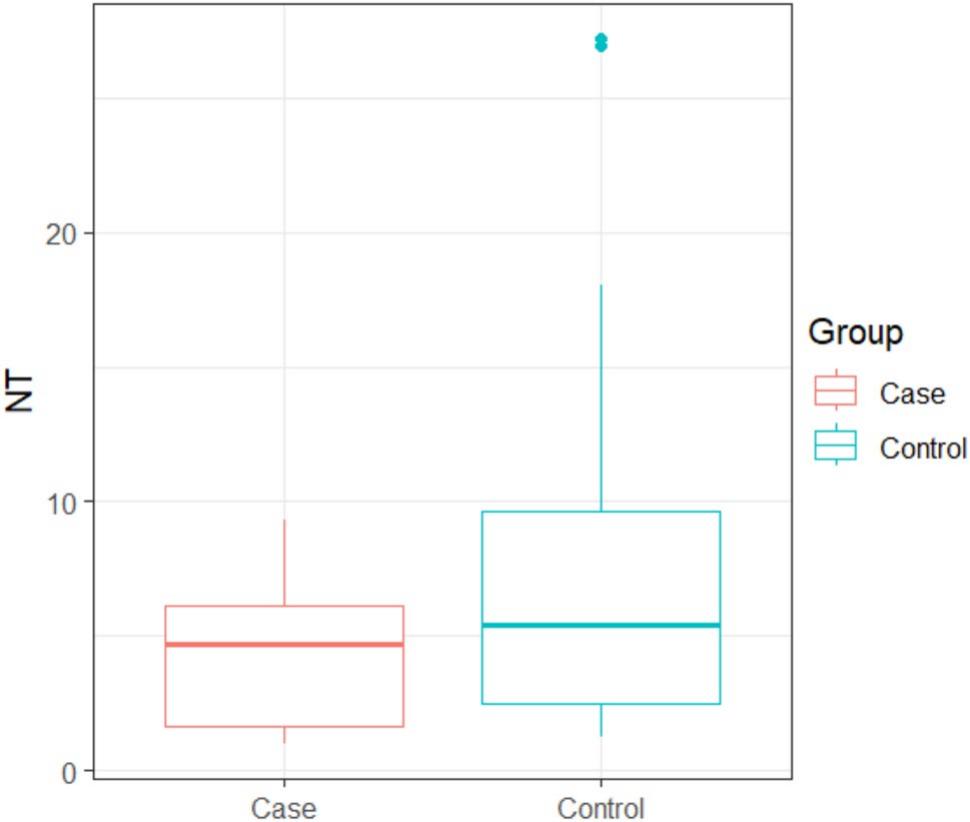
Box plot for NT in both groups (72 h)

NT at 72 h ranged between (0.99 – 9.3) with mean ± SD of (4.5 ± 2.8) in the clindamycin group, while it ranged between (1.23 – 27.2) with mean ± SD of (8.29 ± 7.97) in the control group with a statistically significant difference between the two studied groups (*p* =0.0418), as shown in Table 2 and Fig. 5.

GCS scores at 24 h exhibited a range of (10 - 14) with mean ± SD of (12.41 ± 1.01) in the clindamycin group, while it was (9 - 14) with mean ± SD of (12.5 ± 1.57) in the control group, with no statistically significant difference between the two studied groups at (*p* =0.803).

GCS at 72 h ranged from (10 - 15) with mean ± SD of (14.32 ± 1.13) in the clindamycin group, while it ranged from (11 - 15) with mean ± SD of (14.23 ± 1.31) in the control group. No statistically significant difference was observed between the two studied groups at (*p* =0.724), as shown in Table 2.

**Fig. 6:**
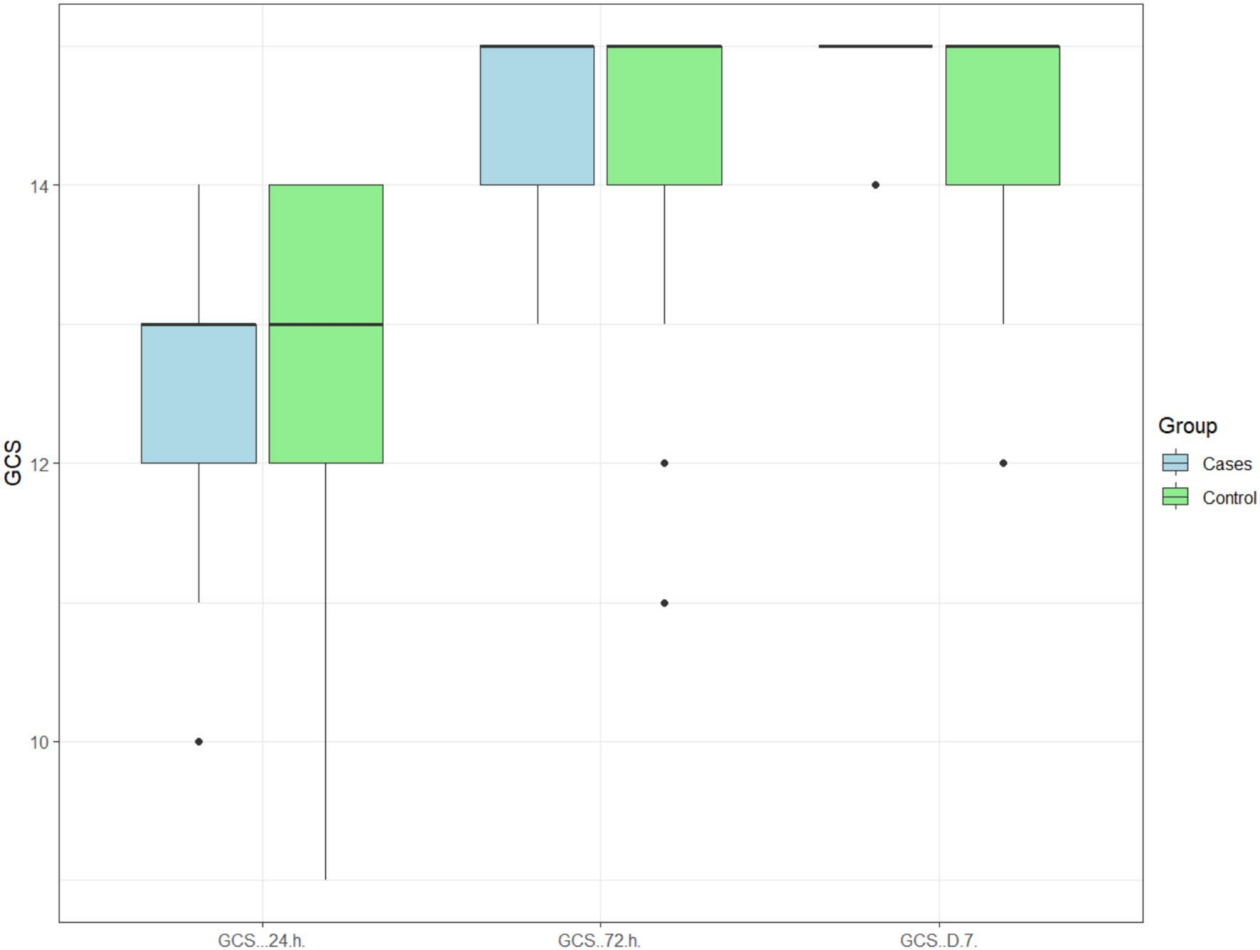
Cluster box plot showing the difference between GCS at 24 h, 72 h, and 7 days between the two studied groups

GCS at 7 days ranged between (14 - 15) with mean ± SD of (14.82 ± 0.6) in the clindamycin group, while it was (12 - 15) with mean ± SD of (14.45 ± 0.91) in the control group. There was no statistically significant difference between the two studied groups at (*p* =0.0881), as shown in Fig. 6.

**Fig. 7:**
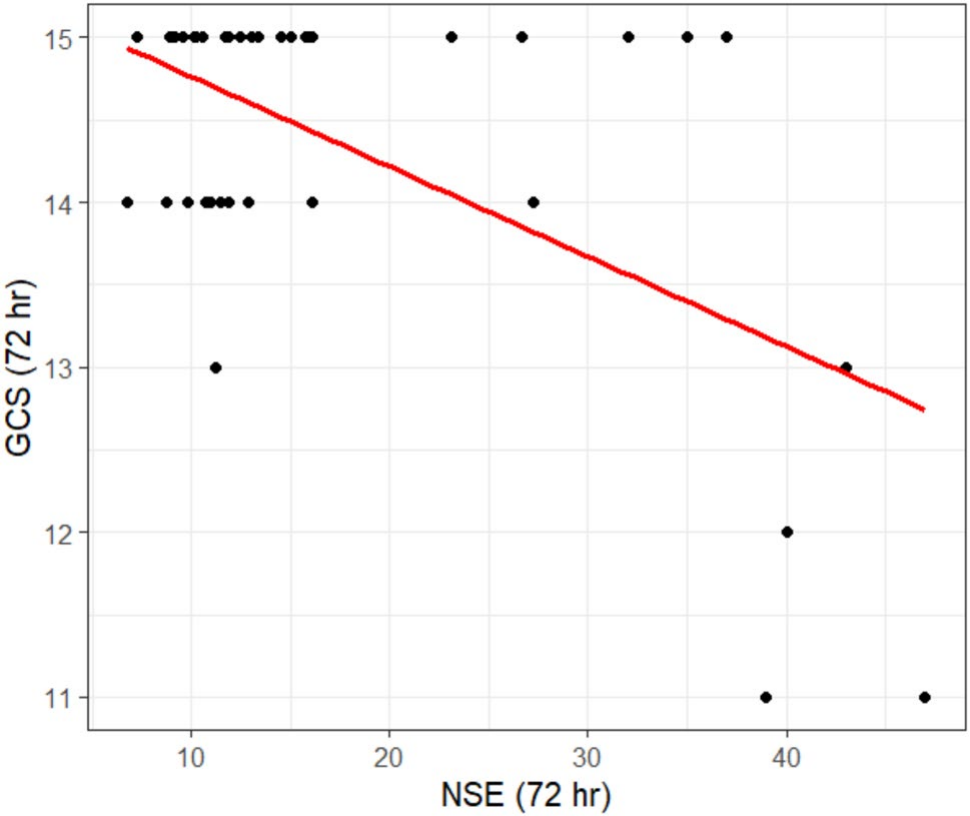
Correlation between NSE at 72 h and GCS at 72 h in the two studied groups

Regarding the correlation between NSE at 24 h and GCS at 24 h, there was a moderately negative correlation that was no statistically significant difference at (r = -0.18, *p* =0.23), as shown in Table 3, while the NSE at 72 h and GCS at 72 h, there was a moderately negative correlation that was statistically significant in all studied patients at (r = -0.31, *p* =0.03), as shown in Table 4 and Fig. 7.

**Table 3:** Correlation between NSE and GCS at 24 h in the two studied groups

NSE (24 h)		
	R	p-value
GCS (24 h)	−0.18	0.23

**Table 4:** Correlation between NSE and GCS at 72 h in the two studied groups

NSE (72 h)		
	R	p-value
GCS (72 h)	−0.31	0.03*

Regarding the distribution of SOFA, the most common grades of SOFA were grade 2 in 15 patients (68.2%) and grade 1 in five patients (22.7%) in the patient group. However, the most common SOFA grades were grade 2 in 14 patients (63.6%) and grade 3 in five patients (22.7%) in the control group. No statistically significant difference was noted between the studied groups in terms of SOFA, as demonstrated in Table 2.

**Fig. 8:**
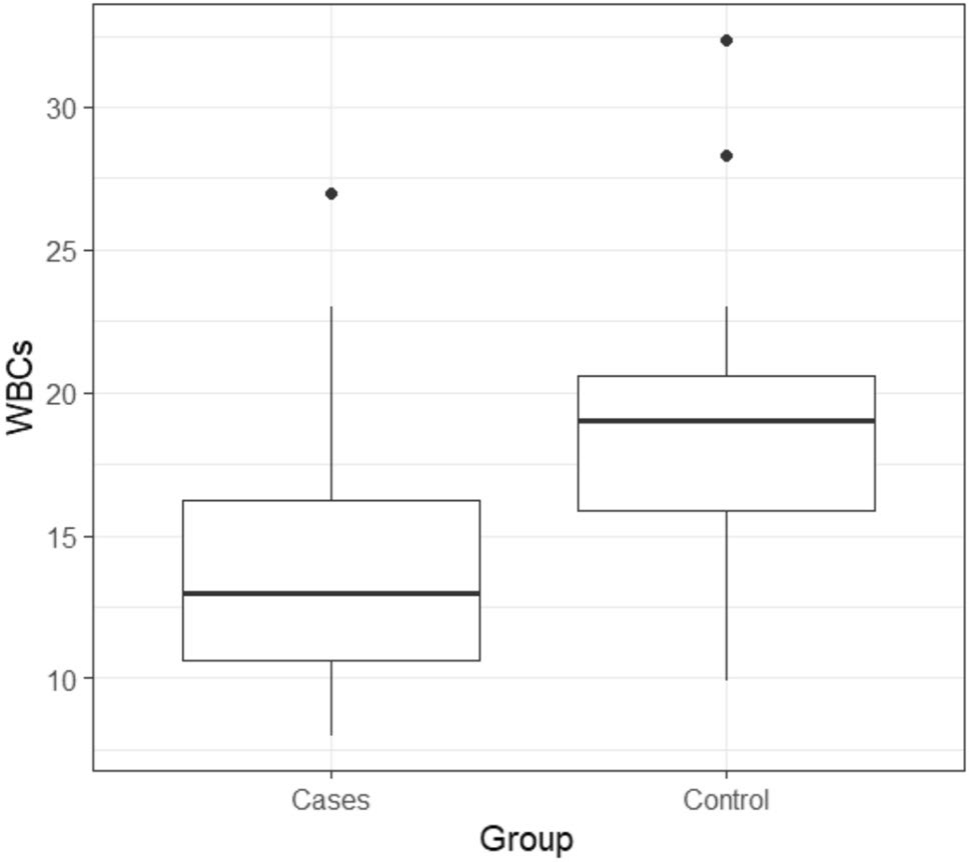
Box plot for WBCs between the two studied groups

Moreover, the WBC decreased in the clindamycin group compared to the control group. WBC exhibited a range of (7.99 – 27) with mean ± SD of (14.58 ± 5.49) in the clindamycin group, while it ranged between (9.9 – 32.38) with mean ± SD of (18.78 ± 5.05) in the control group. There was a statistically significant difference between the two studied groups at (p =0.004), as illustrated in Fig. 8.

**Fig. 9:**
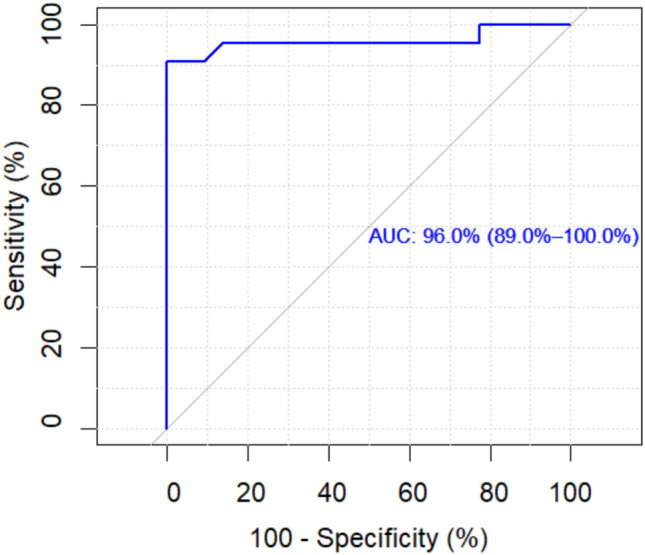
ROC curve of NSE at 72 h between the two studied groups to detect the unhealthy cutoff point

A ROC curve for NSE at 72 h was constructed to detect the unhealthy level, and the corresponding areas under the curve (AUC) were found to be 87.9% (*p*<0.001). The best cutoff value for NSE (72 h) for identifying the unhealthy patients among all studied individuals was <13, where sensitivity was 86.4 % and specificity 95.5 %, as demonstrated in Fig. 9.

## Discussion

The primary aim of the study was the prevention of secondary insults in neurosurgery patients and the protection of their nerves from damage and apoptosis. Currently, the available data provide only symptomatic treatment and prevention of postsurgical infection but with no impact on the progression of secondary injuries following surgeries (Galgano et al. 2017). Recent preclinical studies revealed that clindamycin is a promising alternative to enhance recovery or halt secondary brain injury and neurological necrosis. Accordingly, this study was designed to clinically investigate the benefit of adjunctive clindamycin use in post-neurosurgery patients.

NSE is a valuable and significant diagnostic and prognostic biomarker for acute neuronal cell body injury (Persson et al. 1987). A strong relationship was identified between serum levels of NSE and neurological outcomes (Schmechel et al. 1978). Moreover, NT stimulates mast cells, releasing numerous neurosensitizing and proinflammatory mediators with subsequent exacerbation of neuropathic pain. Also, its altered value indicates that glutamate is not elevated, preventing neuronal excitotoxicity and neural damage. Hence, the effect of clindamycin on the serum levels of NSE and NT was taken as the primary outcome. A similar approach was followed to assess the neuroprotective effects of doxycycline (Mansour et al. 2021), L-carnitine (Mahmoodpoor et al. 2018), and memantine (Radomska-Leśniewska et al. 2016) in TBI patients.

The current study revealed that serum NSE levels measured on day 3 were significantly reduced in neurosurgery patients who received a postoperative combination protocol of clindamycin and ceftriaxone compared with patients receiving ceftriaxone alone. Moreover, a significant difference was observed in the proportion of patients who achieved normalized NT levels, favoring clindamycin. At baseline, a visual difference in the distribution pattern of NSE and NT levels was observed between the two study groups. Despite this heterogeneity, the significantly higher percentage reduction in NSE levels from baseline reported on day 3 in clindamycin-treated patients denotes its potential benefit. This is the first randomized study to examine the possible effects of clindamycin on serum levels of NSE and NT in patients undergoing neurosurgery.

Clindamycin halts the secondary insults that occur after neurosurgery, such as neuroinflammation, brain edema, vasculitis, secondary ischemia, and neural damage and apoptosis during the surgery.

In previous preclinical studies, clindamycin has shown stimulation of the phagocytic activity of mouse macrophages in vivo (Bӧttcher et al., 2004). Other authors have also reported this antibiotic’s stimulation of phagocytic activity in ex vivo and *in vivo* experiments. Furthermore, clindamycin did not exert any effect on protein synthesis or chemotaxis. LPS or concanavalin A-induced lymphocyte proliferation, as well as humoral immunity, was not influenced by clindamycin (Amurrio et al. 1990; Roszkowski et al. 1985).

Additionally, clindamycin may be used for patients with infections and low angiogenesis levels to promote new vessel formation during prolonged repair and healing processes, which could result in long-term improvement (Radomska-Leśniewska, Skopińska-Rȯżewska, and Malejczyk, 2010).

Compared with ceftriaxone, clindamycin reduced the release of lipoteichoic acids from the bacteria into the CSF and the CSF leukocyte count. This led to lower extracellular concentrations of hydroxyl radicals and glutamate in the hippocampal formation, a subsequent reduction of extracellular glycerol levels, and neuronal apoptosis in the dentate gyrus (Zysk et al. 1996), resulting in a decrease in NSE and NT levels. Similarly, a prior study found that serum NSE levels measured on day 7 were significantly reduced in patients with traumatic brain injury (TBI) who received doxycycline (Mansour et al. 2021). Consistently a recent study illustrated that inflammatory biomarkers NSE, neurotensin 3 (NT3), and interleukin-18 (IL-18) levels significantly decreased after treatment with amantadine compared with the placebo group (Werida, R et al., 2025).

Additionally, there is further evidence on the immunomodulatory effects of clindamycin, which was linked to decreased production of TNF-α and inhibition of NF-κB. These effects were seen in a clindamycin derivative with decreased antibacterial activity, suggesting that clindamycin derivatives should be studied as potential therapeutic options for treating painful and inflammatory disorders (Rodrigues et al. 2023). 

This study aimed to investigate the impact of the short-term use of clindamycin on NSE, one of the acute brain injury biomarkers, as well as on NT. The short duration and the small dose used might explain the mild functional neurological outcomes. Higher doses or use for more extended periods may be needed for clindamycin to improve long-term neurological outcomes in patients undergoing neurosurgery significantly.

The positive influence of clindamycin on NSE biomarker serum level was coupled with significantly higher GCS scores in the clindamycin group at the end of the treatment period. Our results revealed a significant negative correlation between NSE and GCS on day 3. This agrees with the findings from previous studies (Meric et al. 2010). The difference in the patient population, as all their enrolled patients had various neurosurgeries with various severities, is attributed to the lack of correlation with our results.

Moreover, the most common grade of SOFA was grade 2 in both the clindamycin and control groups. However, no statistically significant difference regarding SOFA was observed between the studied groups.

The safe pharmacologic profile of clindamycin warrants further clinical trials to focus on its dosing regimen and duration. Additional studies are also needed to determine if the positive effects of clindamycin would be sustained in other neurosurgical procedures.

The current study mainly targeted the exploration, for the first time, of the potential therapeutic opportunity of clindamycin in neurosurgery patients. The neurological outcomes could be affected by confounders such as age, comorbidities, or sex. The small sample size limited this study. The simple randomization in our study hinders the ability to examine how the confounders might potentially impact the study outcomes. Neither the precise mechanism for secondary brain injury nor the optimum regimen for clindamycin use as a neuroprotective drug is known.

## Limitations of the study

The sample size is relatively small, limiting the generalizability of the study’s results. Also, the intervention period was short. A longer follow-up may probably reveal more prominent changes in the levels of both biomarkers. Thus, studies with a larger sample size over extended periods are required to better evaluate the effects of clindamycin on the biomarker levels, facilitating the generalization of findings.

## Conclusion

Adjunctive administration of clindamycin might have a neuroprotective effect in patients after neurosurgery. Clindamycin resulted in a significant reduction of the NSE serum levels, a neuronal damage biomarker, and a decrease in the NT serum level, an inflammatory biomarker. Also, favorable short-term outcomes associated with the GCS scores were noted. Considering the long history of safe use of clindamycin in clinical settings, future larger clinical trials with stratified subsets of participants and long-term outcomes are necessary to confirm these findings. Different dosing regimens are also warranted to explore the potential magnitude of clindamycin’s benefit in halting the neurodegeneration progression in neurosurgery patients.

## Data Availability

The databases used or analyzed during the current study are available from the corresponding author upon reasonable request.

## Abbreviations

CSF: Cerebrospinal fluid
NSE: Neuron-specific enolase
NT: Neurotensin
GCS: Glasgow coma scale
SOFA: Sequential organ failure assessment
ROC: Receiver operating characteristic curve
LPS: Lipopolysaccharide

## References

[CR1] Amurrio C, Lewden S, Nicolas R, Gonzalez P, Cañavate ML, Cisterna R (1990) Effect of treatment with clindamycin, erythromycin, rifamycin or gentamicin on the ingestion capacity of peritoneal macrophages in mice. Pathol Biol (Paris) 38(1):13–18 2407991

[CR2] Barker FG (2007) Efficacy of prophylactic antibiotics against meningitis after craniotomy: a meta-analysis. Neurosurgery 60(5):887–894 17460524 10.1227/01.NEU.0000255425.31797.23

[CR3] Boia R, Ruzafa N, Aires ID, Pereiro X, Ambrósio AF, Vecino E, Santiago AR (2020) Neuroprotective strategies for retinal ganglion cell degeneration: current status and challenges ahead. Int J Molecular Sci 21(7):2262 10.3390/ijms21072262 PMC7177277 32218163

[CR4] Böttcher T, Gerber J, Wellmer A, Smirnov AV, Fakhrjanali F, Mix E, Pilz J, Zettl UK, Nau R (2000) Rifampin reduces production of reactive oxygen species of cerebrospinal fluid phagocytes and hippocampal neuronal apoptosis in experimental Streptococcus pneumoniae meningitis. J Infectious Dis 181(6):2095–2098 10837202 10.1086/315518

[CR5] Bӧttcher T, Ren H, Goiny M, Gerber J, Lykkesfeldt J, Kuhnt U, Lotz M, Bunkowski S, Werner C, Schau I, Spreer A, Christen S, Nau R (2004) Clindamycin is neuroprotective in experimental Streptococcus pneumoniae meningitis compared with ceftriaxone. J Neurochem 91(6):1450–1456 15584921 10.1111/j.1471-4159.2004.02837.x

[CR6] Carapetis JR, Jacoby P, Carville K, Ang SJ, Curtis N, Andrews R (2014) Effectiveness of clindamycin and intravenous immunoglobulin, and risk of disease in contacts, in invasive group A streptococcal infections. Clin Infectious Dis 59(3):358–365 10.1093/cid/ciu304 24785239

[CR7] Castagliuolo I, Wang C-C, Valenick L, Pasha A, Nikulasson S, Carraway RE, Pothoulakis C (1999) Neurotensin is a proinflammatory neuropeptide in colonic inflammation. J Clin Investigation 103(6):843–849 10.1172/JCI4217 PMC408137 10079105

[CR8] Dewan MC, Rattani A, Fieggen G, Arraez MA, Servadei F (2018) Global neurosurgery: the current capacity and deficit in the provision of essential neurosurgical care. Executive summary of the global neurosurgery initiative at the program in global surgery and social change. J Neurosurg 130(4):1055–1064 29701548 10.3171/2017.11.JNS171500

[CR9] Dicou E, Vincent JP, Mazella J (2004) Neurotensin receptor-3/sortilin mediates neurotensin-induced cytokine/chemokine expression in a murine microglial cell line. J Neurosci Res 78(1):92–99 15372498 10.1002/jnr.20231

[CR10] El-Ansary A, Shaker GH, Siddiqi NJ, Al-Ayadhi LY (2013) ‘Possible ameliorative effects of antioxidants on propionic acid/clindamycin-induced neurotoxicity in Syrian hamsters’, Gut Pathogens, 5(32). 10.1186/1757-4749-5-32 PMC3828401 24188374

[CR11] Esposito S, Noviello S, Vanasia A, Venturino P (2004) Ceftriaxone versus other antibiotics for surgical prophylaxis: a meta-analysis. Clin Drug Investigation 24(1):29–39 10.2165/00044011-200424010-00004 17516688

[CR12] Ethiraj R, Thiruvengadam E, Sampath VS, Vahid A, Raj J (2014) Development and validation of stability indicating spectroscopic method for content analysis of ceftriaxone sodium in pharmaceuticals. Int Scholarly Res Notices 2014:278173 10.1155/2014/278173 PMC4897461 27355020

[CR13] Ferraro L, Beggiato S, Tomasini MC, Fuxe K, Tanganelli S, Antonelli T (2011) Neurotensin regulates cortical glutamate transmission by modulating N-methyl-D-aspartate receptor functional activity: an in vivo microdialysis study. J Neurosci Res 89(10):1618–1626 21656844 10.1002/jnr.22686

[CR14] Galgano M, Toshkezi G, Qiu X, Russell T, Chin L, Zhao LR (2017) Traumatic brain injury: current treatment strategies and future endeavors. Cell Trans 26(7):1118–1130 10.1177/0963689717714102 PMC5657730 28933211

[CR15] Goldman R, Bar-Shavit Z, Romeo D (1983) Neurotensin modulates human neutrophil locomotion and phagocytic capability. FEBS Lett 159(1–2):63–67 6873304 10.1016/0014-5793(83)80417-7

[CR16] Haque A, Polcyn R, Matzelle D, Banik NL (2018) New insights into the role of neuron-specific enolase in neuro-inflammation, neurodegeneration, and neuroprotection. Brain Sci 8(2):33 29463007 10.3390/brainsci8020033 PMC5836052

[CR17] Huidobro-Toro JP, Yoshimura K (1983) Pharmacological characterization of the inhibitory effects of neurotensin on the rabbit ileum myenteric plexus preparation. British J Pharmacol 80(4):645–653 10.1111/j.1476-5381.1983.tb10054.x PMC2045053 6152827

[CR18] Iacob G, Iacob S, Cojocaru I (2007) Profilaxia cu antibiotice în neurochirurgie [Prophylactic antibiotics in neurosurgery]. Revista Medico-Chirurgicală a Societăţii De Medici Şi Naturalişti Din Iaşi 111(3):643–648 18293694

[CR19] Kishi K, Hirai K, Hiramatsu K, Yamasaki T, Nasu M (1999) Clindamycin suppresses endotoxin released by ceftazidime-treated Escherichia coli O55:B5 and subsequent production of tumor necrosis factor alpha and interleukin-1β. Antimicrob Agents Chemother 43(3):616–622 10049276 10.1128/aac.43.3.616 PMC89169

[CR20] Lee D, Cho Y, Ko Y, Heo NH, Kang HG, Han S (2021) Neuron-specific enolase level as a predictor of neurological outcome in near-hanging patients: a retrospective multicenter study. PLoS ONE 16(2):e0246898 33566872 10.1371/journal.pone.0246898 PMC7875384

[CR21] Lemaire I (1988) Neurotensin enhances IL-1 production by activated alveolar macrophages. J Immunol 140(9):2983–2988 2834448

[CR22] Lima JE, Takayanagui OM, Garcia LV, Leite JP (2004) Use of neuron-specific enolase for assessing the severity and outcome in patients with neurological disorders. Brazil J Med Biol Res 37(1):19–26 10.1590/s0100-879x2004000100003 14689039

[CR23] Mahmoodpoor A, Shokouhi G, Hamishehkar H, Soleimanpour H, Sanaie S, Porhomayon J, Rasouli F, Nader ND (2018) A pilot trial of l-carnitine in patients with traumatic brain injury: effects on biomarkers of injury. J Critical Care 45:128–132 29454227 10.1016/j.jcrc.2018.01.029

[CR24] Mansour NO, Shama MA, Werida RH (2021) The effect of doxycycline on neuron-specific enolase in patients with traumatic brain injury: a randomized controlled trial. Therapeutic Adv Chronic Dis 12:20406223211024360 10.1177/20406223211024362 PMC8246481 34262678

[CR25] Meric E, Gunduz A, Turedi S, Cakir E, Yandi M (2010) The prognostic value of neuron-specific enolase in head trauma patients. J Emerg Med 38(3):297–301 18499387 10.1016/j.jemermed.2007.11.032

[CR26] Motoyoshi N, Tsutsui M, Soman K et al (2024) Neuron-specific enolase levels immediately following cardiovascular surgery is modulated by hemolysis due to cardiopulmonary bypass, making it unsuitable as a brain damage biomarker. J Artificial Organs 27:100–107 10.1007/s10047-023-01398-9 PMC11126439 37120686

[CR27] Moujalled D, Strasser A, Liddell JR (2021) Molecular mechanisms of cell death in neurological diseases. Cell Death Differ 28:2029–2044 34099897 10.1038/s41418-021-00814-y PMC8257776

[CR28] Nau R, Wellmer A, Soto A, Koch K, Schneider O, Schmidt H, Gerber J, Michel U, Brück W (1999) Rifampin reduces early mortality in experimental Streptococcus pneumoniae meningitis. J Infectious Dis 179(6):1557–1560 10228082 10.1086/314760

[CR29] Nau R, Eiffert H (2002) Modulation of release of proinflammatory bacterial compounds by antibacterials: potential impact on course of inflammation and outcome in sepsis and meningitis. Clinical Microbiology Reviews, 15(1). 10.1128/CMR.15.1.95-110.2002 PMC118062 11781269

[CR30] Patel AM, Periasamy H, Mokale SN (2020) Immunomodulatory dose of clindamycin in combination with ceftriaxone improves survival and prevents organ damage in murine polymicrobial sepsis. Naunyn-Schmiedeberg’s Archiv Pharmacol 393(9):1671–1679 10.1007/s00210-020-01876-4 32383029

[CR31] Persson L, Hårdemark HG, Gustafsson J, Rundström G, Mendel-Hartvig I, Esscher T, Påhlman S (1987) S-100 protein and neuron-specific enolase in cerebrospinal fluid and serum: markers of cell damage in human central nervous system. Stroke 18(5):911–918 3629651 10.1161/01.str.18.5.911

[CR32] Picardi JL, Lewis HP, Tan JS, Phair JP (1975) Clindamycin concentrations in the central nervous system of primates before and after head trauma. J Neurosurg 43(6):717–720 811766 10.3171/jns.1975.43.6.0717

[CR33] Radomska-Leśniewska DM, Skopińska-Różewska E, Malejczyk J (2010) The effect of clindamycin and lincomycin on angiogenic activity of human blood mononuclear cells. Central Eur J Immunol 35:217–222

[CR34] Radomska-Leśniewska DM, Skopińska-Różewska E, Jóźwiak J, Demkow U, Bałan J (2016) Angiomodulatory properties of some antibiotics and Tołpa Peat Preparation. Central Eur J Immunol 41(1):19–24 10.5114/ceji.2016.58312 PMC4829815 27095918

[CR35] Rodrigues FF, Morais MI, Melo ISF et al (2020) Clindamycin inhibits nociceptive response by reducing tumor necrosis factor-α and CXCL-1 production and activating opioidergic mechanisms. Inflammopharmacology 28(2):551–561 31768707 10.1007/s10787-019-00670-w

[CR36] Rodrigues FF, Lino CI, Oliveira VLS et al (2023) ‘A clindamycin acetylated derivative with reduced antibacterial activity inhibits articular hyperalgesia and edema by attenuating neutrophil recruitment NF-κB activation and tumor necrosis factor-α production. Int Immunopharmacol 122:110609 37429145 10.1016/j.intimp.2023.110609

[CR37] Roszkowski W, Ko HL, Roszkowski K, Jeljaszewicz J, Pulverer G (1985) Antibiotics and immunomodulation: effects of cefotaxime, amikacin, mezlocillin, piperacillin and clindamycin. Med Microbiol Immunol 173(5):279–289 3969058 10.1007/BF02124944

[CR38] Schmechel D, Marangos PJ, Brightman M (1978) Neurone-specific enolase is a molecular marker for peripheral and central neuroendocrine cells. Nature 276(5690):834–836 31568 10.1038/276834a0

[CR39] Schoerkhuber W, Kittler H, Sterz F, Behringer W, Holzer M, Frossard M, Spitzauer SA, Laggner AN (1999) Time course of serum neuron-specific enolase. Stroke, 30(8). 10.1161/01.str.30.8.1598 10436107

[CR40] Tan YC, Gill AK, Kim KS (2015) Treatment strategies for central nervous system infections: an update. Exp Opinion on Pharmacotherapy 16(2):187–203 10.1517/14656566.2015.973851 25328149

[CR41] Waraich M, Ajayan N (2024) Clinical neuroprotection and secondary neuronal injury mechanisms. Anaesthesia & Intensive Care Med 25(1):16–22

[CR42] Werida R, ElMalky M, Shama M, Ghoneim A (2025) Impact of amantadine on inflammatory biomarkers in traumatic brain injury patients: a randomized controlled trial. J Adv Med Pharmaceutical Res 6(1):79–86

[CR43] Wright JM, Collier B (1976) Characterization of the neuromuscular block produced by clindamycin and lincomycin. Canadian J Physiol Pharmacol 54(6):937–944 10.1139/y76-130 191168

[CR44] Zysk G, Brück W, Gerber J, Brück Y, Prange HW, Nau R (1996) Anti-inflammatory treatment influences neuronal apoptotic cell death in the dentate gyrus in experimental pneumococcal meningitis. J Neuropathol Exp Neurol 55(6):722–728 8642398 10.1097/00005072-199606000-00006

